# Cervical and Lumbar Disc Arthroplasty: A Review of Current Implant Design and Outcomes

**DOI:** 10.3390/bioengineering9050227

**Published:** 2022-05-23

**Authors:** Ian J. Wellington, Cameron Kia, Ergin Coskun, Barrett B. Torre, Christopher L. Antonacci, Michael R. Mancini, John P. Connors, Sean M. Esmende, Heeren S. Makanji

**Affiliations:** 1 Department of Orthopaedics, University of Connecticut, Farmington, CT 06032, USA; iwellington@uchc.edu (I.J.W.); coskun@uchc.edu (E.C.); btorre@uchc.edu (B.B.T.); cantonacci@uchc.edu (C.L.A.); mimancini@uchc.edu (M.R.M.); jconnors@uchc.edu (J.P.C.); 2 Department of Orthopedics, Hartford Healthcare, Hartford, CT 06106, USA; sesmende@oahctmd.com (S.M.E.); hmakanji@oahctmd.com (H.S.M.)

**Keywords:** arthroplasty, fusion, spine, disc, implant, biomechanics

## Abstract

While spinal disc pathology has traditionally been treated using fusion-based procedures, recent interest in motion-preserving disc arthroplasties has grown. Traditional spinal fusion is associated with loss of motion, alteration of native spine kinematics, and increased risks of adjacent segment disease. The motion conferred by disc arthroplasty is believed to combat these complications. While the first implant designs resulted in poor patient outcomes, recent advances in implant design and technology have shown promising radiographic and clinical outcomes when compared with traditional fusion. These results have led to a rapid increase in the utilization of disc arthroplasty, with rates of cervical arthroplasty nearly tripling over the course of 7 years. The purpose of this review was to discuss the evolution of implant design, the current implant designs utilized, and their associated outcomes. Although disc arthroplasty shows significant promise in addressing some of the drawbacks associated with fusion, it is not without its own risks. Osteolysis, implant migration, and the development of heterotopic ossification have all been associated with disc arthroplasty. As interest in these procedures grows, so does the interest in developing improved implant designs aimed at decreasing these adverse outcomes. Though they are still relatively new, cervical and lumbar disc arthroplasty are likely to become foundational methodologies for the treatment of disc pathology.

## 1. Introduction

The traditional approach to addressing degenerative cervical and lumbar pathology is to utilize fusion-based procedures. This often involves discectomy and placement of a static implant and is associated with several potential complications including adjacent segment disease and loss of spinal motion [1,2]. Both cervical and lumbar disc arthroplasties were developed as an alternative to fusion with the goal of preserving native spine motion while addressing intervertebral pathology.

Cervical and lumbar disc arthroplasty began in the 1960s [3]. Both of these procedures utilized a metallic sphere placed within the annulus fibrosis which aimed to allow for maintained motion after the removal of a symptomatic disc. While initially successful, both procedures suffered from high complication rates and implant subsidence [4,5,6]. Since that time, advancements in technology have allowed for the development of a multitude of implant designs which have shown superior clinical outcomes when compared with the initial designs. These new designs offer various degrees of constraint, are composed of different materials, and employ a variety of mechanisms to promote osteointegration [7,8]. While a wide variety of artificial disc models have been developed, there remain major challenges for disc replacement including optimization of implants to reduce wear, prevent implant migration, and prevent continued degenerative changes affecting the bony integrity of the spine.

This review aimed to discuss the history of both cervical and lumbar disc replacement technology, explain how these procedures are performed, and address the current clinical and biomechanical data surrounding these procedures. This review also aimed to highlight current implant designs and their rationale. Ultimately, while both cervical and lumbar disc replacements are still in their relative infancies, both are likely to become commonplace for the management of cervical and lumbar disc pathology.

## 2. Cervical Disc Arthroplasty

### 2.1. Early Development

Cervical disc arthroplasty (CDA) was first performed in the 1960s in Sweden when Ulf Fernström implanted a stainless steel ball-bearing prosthesis in the cervical spine [3]. Hjalmar Reitz and Mauritius Joubert, who had previously met with Fernström to observe his technique, published a series of 75 CDAs using the same ball-bearing prosthesis for the treatment of intractable headaches and neck pain, with initially promising outcomes [9]. However, both Fernström and Reitz ultimately demonstrated poor results with these prostheses with high rates of segment hypermobility and subsidence at 7 years of follow-up [4]. These outcomes initially hindered the progression of CDA.

In the 1980s, however, interest in CDA was renewed. B.H. Cummins in the United Kingdom developed the Cummins–Bristol Disc, a two-pieced ball-and-socket style device composed of stainless steel with anterior screws placed into the superior and inferior endplates [4]. Cummins published a series of 22 joints replaced in 20 patients with this implant, of which, 3 had screw pullout, 3 had persistent dysphagia, 1 had screw breakage, and 1 had joint subluxation [10]. However, 16 of the 20 patients reported long-lasting improvement in their preoperative pain. With this renewed interest in CDA, and modernized techniques and materials, the development of new arthroplasty implants continued.

### 2.2. Modern Implant Design

Modern CDA implants can be categorized into two major categories: semi-constrained and unconstrained. Unconstrained implants have no mechanical limitation to motion. While this increases mobility, it may increase the risk of instability. Semi-constrained implants have physical motion stops outside of the normal physiologic range, thus conferring the benefits of unconstrained mobility, with greater stability if an implant undergoes excessive motion [7]. Additionally, implants can differ in their material composition (stainless steel, cobalt, or titanium), endplate surface (keels, spikes, porous coating, or screws), or type of articulation (ball-and-socket versus saddle) [7].

**Figure 1:**
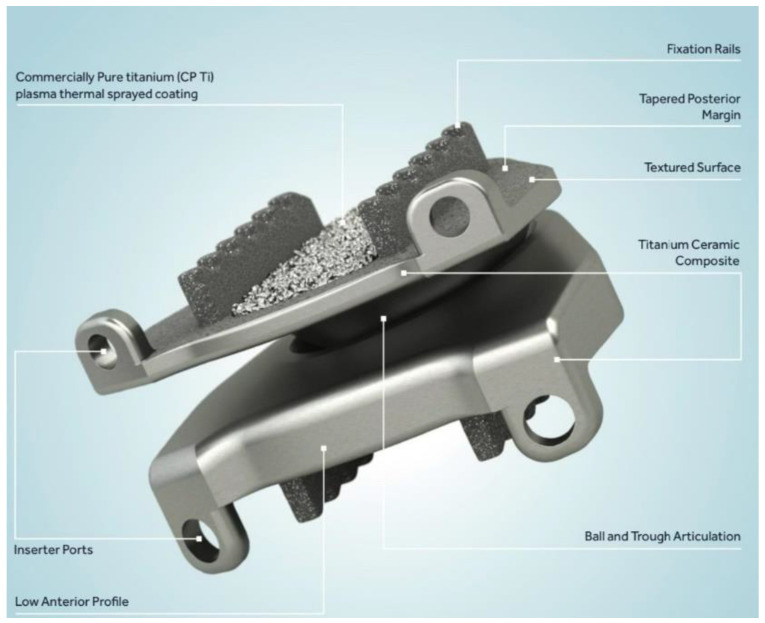
The Prestige cervical disc arthroplasty implant. It is composed of two metal endplate pieces with a ball and trough style articulation. Printed with permission from Medtronic PLC (Minneapolis, MN, USA).

**Figure 2:**
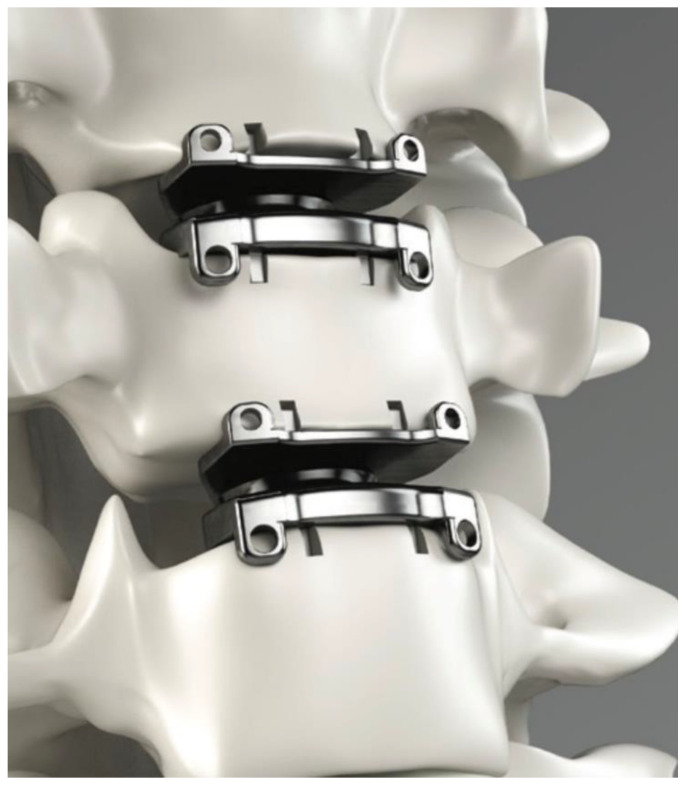
Cervical disc arthroplasty implants placed in two consecutive levels. Printed with permission from Medtronic PLC (Minneapolis, MN, USA).

The current available unconstrained implants are the Prestige, the Discover, the Bryan, the M6, and the Mobi-C. The Prestige is a two-piece, metal-on-metal device composed of either titanium or stainless steel which has shown superior overall patient-reported success rates (74.9%) when compared with traditional anterior cervical decompression and fusion (ACDF) (63.2%) at 84 months (Figure 1) [11]. The Discover implant is a ball-and-socket style implant composed of titanium alloy with an ultra-high molecular weight polyethylene (UHWMPE) insert and utilizes both keels and a plasma spray porous coating on the endplates to encourage bony ingrowth. A review of studies comparing the Discover system to ACDF found that the Discover was associated with shorter operative time and better postoperative range of motion, with no difference in neck disability index scores or adverse events [12]. The Bryan artificial disc is a single unit device with two titanium alloy endplates and a polyurethane center with a porous coated surface. At 24 months, patients who underwent arthroplasty with the Bryan system showed significant improvements in neck disability index (NDI) and neck pain scores [13]. The M6 disc is a single-piece titanium alloy that contains a polycarbonate urethane polymeric center surrounded by polyethylene woven fiber. This recreates the native annulus fibrosis and nucleus pulposus and allows for flexion, extension, lateral bending, and rotational motion. Finally, the Mobi-C disc is a three-pieced implant with cobalt chrome endplates and a UHMWPE center with both teeth and a hydroxyapatite spray coating on each endplate. Both the M6 and the Mobi-C have shown similarly significant improvements in patient-reported outcome measures compared with patients’ baselines [14]. The Mobi-C and the Prestige are currently the only arthroplasty systems that are approved by the Food and Drug Administration (FDA) for two-level disc replacement (Figure 2) [15].

The CerviCore, Prodisc-C, Porous Coated Motion (PCM), and SECURE-C are all semi-constrained liners. The CerviCore is a two-piece, metal-on-metal, saddle-shaped implant with both keels and spikes on each endplate. At 2 years, patients treated with CerviCore implants showed greater improvements in Worse Arm Visual Analogue Scale (VAS) scores and fewer device-related surgical interventions compared with those treated with ACDF [16]. The Prodisc-C is a two-piece, ball-and-socket implant composed of cobalt chrome alloy and an UHMWPE insert with a porous plasma spray titanium coating on the endplates which has demonstrated a greater reduction in neck pain intensity and frequency when compared with ACDF at 5 years [17]. The PCM is a two-piece cobalt chrome alloy device with a UHMWPE core with a broad radius of curvature to allow for increased lateral endplate support. As its name suggests, it has a titanium calcium phosphate coating to encourage bony ingrowth. The PCM implant has shown fewer device-related adverse events at 2 and 7 years and decreased rates of adjacent level degeneration compared with ACDF [18]. Finally, the SECURE-C is a three-piece implant composed of two cobalt chrome endplates and an UHMWPE center with a porous plasma spray coating and serrated keels on each endplate. The superior aspect of the UHMWPE center is spherical while the inferior aspect is cylindrical, this selectively constrains the articular motion with the hope of better representing the natural motion of the spine. At 24 months, patients treated with the SECURE-C demonstrated similar improvements in both NDI and VAS scores when compared with ACDF with fewer rates of secondary surgical interventions [19].

### 2.3. Surgical Technique

CDA is indicated for patients with cervical disc disease at one or two intervertebral levels in the cervical spine who have failed conservative therapy and are between the ages of 20 and 70 years old. Contraindications include, but are not limited to, three or more levels of pathology, instability, adjacent level of fusion, facet joint degeneration, or severe spondylosis [20].

The patient is positioned supine on a radiolucent table with the neck in the neutral position [7,21]. Intra-operative radiographs are obtained to the appropriate cervical level [21]. The anterior cervical spine is accessed using the standard Smith–Robinson approach [7,21,22]. After incising the skin, the subcutaneous fat and platysma are dissected to identify the superficial fascia. The dissection continues in the plane between the sternocleidomastoid and carotid sheath laterally and the trachea and esophagus medially. The prevertebral fascia is then incised and the longus coli muscles on either side of the midline spine may be retracted. Radiographs are then taken to ensure that the incision is centered on the correct level.

Distraction pins may be placed in the mid-vertebral bodies above and below the disc. Gentle distraction is then applied to these pins to allow greater access to the disc space. Following the incision of the annulus, the disc is removed without damaging the bony endplate, as doing so may increase the risk of implant subsidence.

After the pathologic disc is removed and the vertebral endplates are prepared, implant trailing is performed. Using trial disc devices, the appropriate implant size should be selected under fluoroscopic visualization. The actual implant is placed in the disc space in the correct superior–inferior orientation on lateral fluoroscopy to determine the depth and appropriate central positioning is confirmed on the AP view.

### 2.4. Clinical Outcomes

From 2006 to 2013, the rate of ACDFs and CDAs in the United States rose by 5.7% and 190%, respectively [22]. The number of single-level CDAs for every 100 ACDFs increased from 5.6 in 2009 to 28.8 in 2017, with the largest increases occurring after 2013 [23]. This adoption of CDAs into clinical practice may, in part, be explained by the expanding literature supporting CDA outcomes. In 2021, a meta-analysis by Peng et al. concluded that the overall success rate of CDA was approximately double that of ACDF (OR 1.91; 95% CI (1.73–2.11); *p* = 0.000) with improved outcomes in the short-term (1–3 years), mid-term (4–6 years), and long-term (7+ years) results [24]. The overall success rate was defined as a composite of improved NDI, neurological status, and disc height without implant-related adverse events or subsequent surgical procedures [11]. This suggests that CDI may result in greater patient outcomes when compared with ACDF at all time points.

One reason for the development of CDA was to provide an alternative to ACDF that preserves joint mobility to reduce adjacent segment disease (ASD) [25]. Studies have demonstrated that CDA preserves the mobility of both the operative and upper/lower adjacent cervical segments [26,27,28,29]. This preservation of cervical mobility has been shown to reduce adjacent intradiscal pressures and may prevent accelerated ASD compared with ACDF [30,31]. Given that preventing ASD development is one of the primary goals of CDA, these results are promising.

To date, the literature supports equivalent or decreased rates of ASD following CDA compared with ACDF [27,32,33]. Two recent meta-analyses have demonstrated lower rates of ASD and reoperations following CDA versus ACDF [32,34]. Xu et al. analyzed results of 2632 patients and found lower ASD rates (OR 0.6; 95% CI (0.38–0.73), *p* < 0.00001) and lower reoperation rates (OR 0.52; *p* < 0.001) [32]. Similarly, Zhu et al. analyzed results from 3235 patients and demonstrated lower ASD rates (risk ratio 0.57, 95% CI (0.37–0.87), *p =* 0.009) and lower adjacent segment reoperation rates (risk ratio 0.47, 95% CI (0.32–0.70), *p =* 0.0002) [34]. The reduced rates of ASD have been demonstrated in short-term and long-term outcomes up to 7 years [33,35]. Although reoperation rates for CDA and ACDF vary by study, Chang et al. performed a review of the literature and reported reoperation rates of 3.1% and 6.0% for CDA and ACDF, respectively [36].

The literature also supports favorable outcomes for CDA using measures other than ASD. Compared with ACDF, CDA was shown to have higher overall success (78.6% vs. 62.7%), NDI scores (87.0% vs. 75.6%), and neurological success (91.6% vs. 82.1%) [37]. Several studies also demonstrated that patients with CDA return to work faster than patients with ACDF (range, 13–20 days) [38,39,40]. Lastly, rates of dysphagia are also lower in the short-term after CDA (OR 0.68; 95% CI (0.5–9.1); *p =* 0.01); however, there are no significant differences in the mid- and long-term rates [24]. Furthermore, CDA has been demonstrated to be more cost-effective than ACDF with 5-year costs for CDA estimated at USD 102,274 while ACDF estimated costs were USD 119,814 [41].

While the clinical outcomes of CDA are promising, the procedural complications cannot be overlooked. One of the more common complications is heterotopic ossification (HO), or the formation of bone outside the skeletal system. Following CDA, HO can cause bridging ossification between vertebral end plates, resulting in fusion, thus defeating the purpose of CDA. The rates of HO vary within the literature, ranging from 10–37% [30,40,42,43]. However, it should be noted that Li et al. found an increased rate of HO in patients with spondylosis and concluded that HO may be a result of the degenerative process rather than a complication of CDA [44]. HO has also been shown to increase with time and can appear late in the postoperative process [45]. Increased age and male sex are risk factors for the development of HO [46].

Osteolysis is another common complication following CDA. Rates of osteolysis have been reported in over 50% of cases [42]; however, although it is common, osteolysis is rarely symptomatic and does not often require revisional procedures [43]. Contrary to the progressive nature of HO, osteolysis usually presents within the first year and rarely advances afterward [47,48,49]. If osteolysis does progress, device subsidence into the vertebral endplates can occur. While subsidence has been reported in up to one third of cases, only 3% are symptomatic, presenting most often with pain during motion [50,51].

## 3. Lumbar Disc Arthroplasty

### 3.1. Early Development

Similar to the first cervical disc replacement, the first lumbar disc arthroplasty (LDA) was performed by Ulf Fernström [3]. He utilized a larger version of the implants he used in the cervical spine: stainless steel balls placed between the vertebrae and contained by the annulus fibrosus. As with the cervical arthroplasties, these lumbar patients suffered from high rates of subsidence, with 88% demonstrating loss of intervertebral height at 7-year follow-up. The next iteration of LDA was performed by Fassio and Ginestie in the 1970s. They utilized a silastic ball with a horseshoe-shaped plateau to combat subsidence, however, at 4 years, all patients demonstrated subsidence of the implant into the vertebral bodies [5,6].

In the early 1980s, Chellnack and Buttner-Janz developed the SB Charité, the first commercially available lumbar implant which was composed of two metal endplates and a UHMWPE sliding core [6,47]. The free-floating nature of the polyethylene core allowed the center of rotation of the implant to shift anteriorly and posteriorly through extension and flexion, respectively. This was then followed in the late 1980s by the ProDisc, which contained a UHMWPE liner that was connected to the inferior metal endplate which was then articulated with the superior metal endplate [6]. While the ProDisc did have a larger endplate relative to the initial design of the SB Charité to avoid subsidence, the polyethylene core was locked into the inferior baseplate which prevented the restoration of native motion.

### 3.2. Modern Implant Design

**Figure 3:**
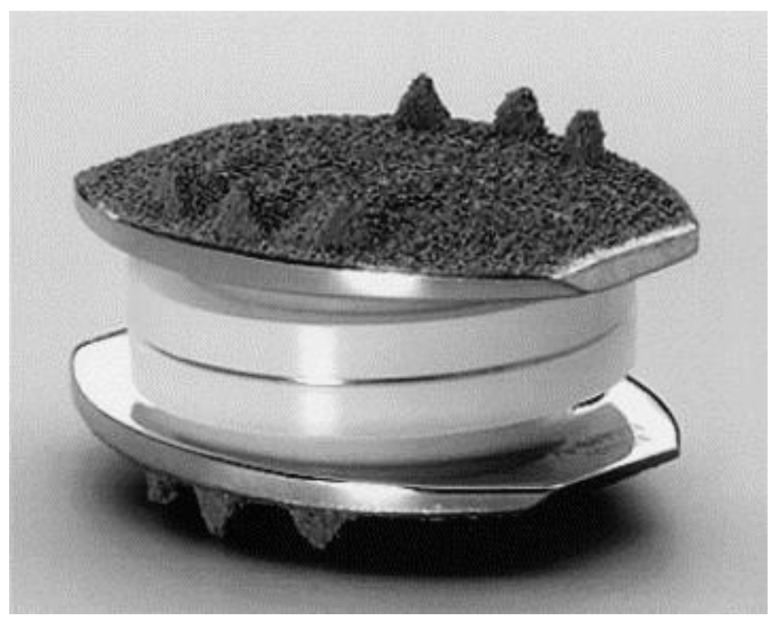
Charité III prosthesis (Depuy Spine, Rynam, MA, USA) composed of two cobalt chromium endplates with a UHMWPE sliding core. Reprinted/adapted with permission from Ref. [48]. 2005, Elsevier.

Much like cervical disc replacement implants, lumbar implants can be categorized as either semi-constrained or unconstrained. Currently, there is one unconstrained implant available, the Charité III (Figure 3). This is the third generation of the original SB Charité implant; however, the modern version has wider endplates to decrease the risks of subsidence. The endplates are made of cobalt chrome and there is a UHMWPE core that sits unconstrained between the metallic endplates. This mobile design allows for sliding during the range of motion [8]. Lu et al. in 2015 published 11-year outcomes of the Charité III implant and found that 28 of 32 patients reported successful outcomes, with 1 patient requiring reoperation for adjacent segment disease and 1 who sustained a pedicle fracture [52]. They also reported a significant improvement in both VAS and Oswestry disability index (ODI) scores in these patients.

The majority of LDA implants are semi-constrained. The Prodisc II, the modern version of the original ProDisc, is composed of two cobalt chrome endplates with a UHMWPE insert that is semi-constrained. Each endplate has both a single serrated keel and two lateral pegs for stability. A study of 53 patients treated with the ProDisc II showed significant improvements in VAS and ODI scores, with similar clinical results in single- and multi-level surgeries [49]. Of these patients, 9% had postoperative complications and 6% required reoperation. Another semi-constrained implant is the Maverick disc. It is made of two cobalt chrome endplates with a concave superior endplate and a convex inferior endplate. Compared with traditional anterior lumbar interbody fusion (ALIF), patients treated with the Maverick disc had significantly greater improvements in the ODI scores at 1, 2, and 5 years with fewer device-related adverse events [53].

Another semi-constrained implant, the ActivL disc, is made up of two cobalt chrome endplates with a titanium and microscopic dicalcium phosphate coating on each endplate [54]. It contains an UHMWPE inlay which allows for translation only in the anterior-to-posterior plane. A study looking at two-year outcomes for patients treated with the ActivL versus the ProDisc or Charité implants found that those who received the Maverick were significantly more likely to achieve a composite successful outcome (composed of >15 points on the ODI, improved neurologic status, improved range of motion, lack of device failure, and lack of device-related complications) when compared with those who received one of the other implants [55]. Finally, the Mobidisc is a cobalt chrome molybdenum implant that contains a semi-constrained UHMWPE insert that articulates with a flat surface on the inferior endplate and a spherical surface on the superior endplate, which allows for both translational and rotational motion. At 2-year follow-up, patients treated with the Mobidisc reported significant improvements in ODI and VAS scores [56]. Many investigational products also exist, with mounting interest in implants that can be introduced via either a posterolateral or lateral approach rather than the more invasive anterior approach that is currently used.

### 3.3. Surgical Technique

LDA is indicated for the treatment of discogenic backpain and disc herniation in the lumbar spine. Contraindications include, but are not limited to, facet arthrosis, central or lateral recess stenosis, spondylolisthesis or spondylolysis, instability, pseudoarthrosis, and scoliosis [57].

LDA is generally performed via an anterior midline approach. The patient is positioned supine on a radiolucent table. A skin incision is made lateral to the umbilicus and carried down until the rectus sheath is encountered [58]. The rectus sheath is incised and dissection is carried through the transversalis fascia then into the retroperitoneal space. Blunt dissection is performed until the psoas is encountered, with care taken to avoid the ureter. The anterior lumbar spine is encountered medial to the psoas. During the approach to the disc space, visualization of the iliac vessels, as well as the iliolumbar artery at L4–5 and median sacral artery at L5-S1 is critical to prevent vascular injury.

After exposing the disc space, the disc is excised and removed, and the vertebral endplates are prepared for instrumentation [59]. Care must be taken not to violate the endplates as this can cause an increased risk of implant subsidence [47,58]. After trial implants are used to determine the appropriate final implant size, the disc replacement implant is inserted into the intervertebral space with the appropriate superior–inferior orientation.

The need to excise the anterior longitudinal ligament (ALL), which provides an anterior restraint not only to extension but also to axial rotation, is a significant disadvantage of the anterior approach [60,61]. It has been demonstrated that resection of the ALL causes segmental instability, hypermobility, and increased stress of the facet joints which may lead to facet arthrosis at either the instrumented level or the adjacent levels [61]. Numerous complications such as sympathetic dysfunction, vascular injury, somatic neural injury, sexual dysfunction, prolonged ileus, wound dehiscence, deep vein thrombosis, and bowel injury have been reported in relation to the anterior approach [62].

### 3.4. Clinical Outcomes

LDA for the treatment of disc pathology was not as rapidly accepted by surgeons as it was in the cervical spine, owing largely due to fewer lumbar implants with approval from the Food and Drug Administration (FDA) [47]. Clinical outcomes following LDA have been promising. Guyer et al. conducted a randomized multicenter prospective study comparing two LDA implant systems with 5 years of follow-up. They reported significantly improved VAS and ODI scores in both groups compared with preoperative scores [63]. They did, however, report an 11% reoperation rate for both implants, a majority of which were posterior decompressions for stenosis. Similar improvements in VAS and ODI scores have been repeatedly demonstrated [52,64]. Radiographic parameters have also been promising for LDA, with a postoperative segmental range of motion reported to be between 2 and 14.6° [65,66]. Heterotopic ossification, much as it is in the cervical spine, is a major concern for LDA. A wide range (between 1.6 and 85%) of rates of HO after LDA have been reported [55,67]. Additionally, multilevel LDA may also confer increased risks of complications when compared with single-level arthroplasty. Siepe et al. found that concomitant L4-L5 and L5-S1 LDA resulted in significantly greater postoperative facet joint pain when compared with single-level L5-S1 LDA [68]. Adjacent segment disease has been shown to be less prevalent following LDA compared with fusion. A systematic review by Harrop et al. found that adjacent segment disease occurred in 14% of fusion patients compared with 1% of LDA patients [69]. As with CDA, the reduction in ASD rates is the main goal with disc arthroplasty, making these results promising.

When compared with lumbar fusion, LDA has demonstrated variable outcomes. Jacobs et al. reported that lumbar disc replacement was superior to lumbar fusion with regard to ODI, VAS back pain, patient satisfaction, implant motion, and subsidence. The authors reported no significant difference between lumbar disc replacement and lumbar fusion for leg pain, blood loss, radiographic loosening, and adjacent segment and facet joint degeneration [70]. A meta-analysis conducted by Yajun et al. included five randomized controlled trials involving 837 patients that demonstrated that lumbar disc replacement patients had significantly higher satisfaction scores compared with the lumbar fusion group at the 2-year follow-up. At 5 years, the authors reported no significant differences between the lumbar disc replacement and lumbar fusion groups [71]. A randomized multicenter prospective study following 304 patients for two years found that LDA demonstrated greater improvements in ODI and VAS when compared with fusion with similar rates of subsidence and revision surgery [72,73]. However, Zigler et al. reported equivalent improvements in VAS and ODI scores for LDA versus fusion [74]. Interestingly, cost analysis comparisons between LDA and fusion found no significant difference in the estimated costs of these procedures; however, these results were determined using an assumed equivalent complication rate, which may not reflect the true outcomes of these procedures [75].

## 4. Conclusions

Degenerative disc pathology remains an ever-present cause of pain and disability for those who suffer from it. While the previous methodologies for addressing this pathology, namely fusion, were effective, they were not without limitations. Loss of motion and risk of adjacent segment disease were potential causes of further disability amongst these patients. Disc arthroplasty was developed as a means of addressing these previous morbidities. While arthroplasty initially demonstrated poor results, as technologies advanced, and implant designs improved, so did patient outcomes.

Both cervical and lumbar disc arthroplasties offer promising solutions to the problems associated with spinal fusion. While both are in their relative infancies, they have shown favorable results with significant improvements in patient outcome measures and decreased rates of adjacent segment disease. While disc arthroplasty is not without its own associated adverse events, such as implant migration, osteolysis, and heterotopic ossification, implant design continues to improve to help combat these. While the overall rates of disc arthroplasty are dwarfed by the rates of fusion for the treatment of disc pathology, as implant design improves and clinical studies continue to demonstrate promising outcomes, it is expected that the rates of arthroplasty will continue to increase. As disc arthroplasty becomes increasingly common, further research will be needed to determine rare adverse outcomes. Additionally, further research in this field is needed regarding implant design, with the goal of optimizing these designs to increase patient satisfaction and maximization of postoperative function.

### Limitations

This review is not without limitations. Firstly, it may not have discussed all available implants, specifically implants that are in their infancy of design or recently on the market. Additionally, there is a risk of bias in how each implant is presented in the review. Finally, current literature discussing disc arthroplasty is rife with potential study bias. Potential conflicts of interest are estimated to be present in 44.9% of CDA literature and in 57% of LDA literature [76,77]. This magnitude of potential industry influence over the current literature calls into question our current understanding of outcomes following these procedures.
